# Submaximal exercise testing with near-infrared spectroscopy in Myalgic Encephalomyelitis/Chronic Fatigue Syndrome patients compared to healthy controls: a case–control study

**DOI:** 10.1186/s12967-015-0527-8

**Published:** 2015-05-20

**Authors:** Ruth R Miller, W Darlene Reid, Andre Mattman, Cristiane Yamabayashi, Theodore Steiner, Shoshana Parker, Jennifer Gardy, Patrick Tang, David M Patrick

**Affiliations:** School of Population and Public Health, British Columbia Centre for Disease Control, University of British Columbia, 655 West 12th Avenue, Vancouver, BC V5Z 4R4 Canada; Department of Physical Therapy, University of Toronto, 160-500 University Avenue, Toronto, ON M5G 1V7 Canada; Adult Metabolic Disease Clinic, Vancouver General Hospital, Level 4, 2775 Laurel Street, Vancouver, BC V5Z 1M9 Canada; Muscle Biophysics Laboratory, Department of Physical Therapy, University of British Columbia, 828 West 10th Avenue, Vancouver, BC V5Z 3P1 Canada; Department of Medicine, Vancouver General Hospital, University of British Columbia, Vancouver, BC V5Z 3J5 Canada; Centre for Health Evaluation and Outcome Sciences, 588-1081 Burrard Street, Vancouver, BC V6Z 1Y6 Canada; British Columbia Centre for Disease Control, 655 West 12th Avenue, Vancouver, BC V5Z 4R4 Canada

**Keywords:** Near-infrared spectroscopy (NIRS), Myalgic Encephalomyelitis/Chronic Fatigue Syndrome (ME/CFS), Tissue oxygen utilization

## Abstract

**Background:**

Myalgic Encephalomyelitis/Chronic Fatigue Syndrome (ME/CFS) is a debilitating illness. Symptoms include profound fatigue and distinctive post-exertional malaise (PEM). We asked whether a submaximal exercise test would prove useful for identifying different patterns of tissue oxygen utilization in individuals with ME/CFS versus healthy subjects. Such a test has potential to aid with ME/CFS diagnosis, or to characterize patients’ illness.

**Methods:**

A case–control study of 16 patients with ME/CFS compared to 16 healthy controls completing a 3-min handgrip protocol was performed. Response was measured using near-infrared spectroscopy, resulting in measurements of oxygenated (O_2_Hb) and deoxygenated hemoglobin (HHb) over wrist extensors and flexors. Changes in O_2_Hb (delta (d)O_2_Hb) and HHb (dHHb) absorbance between the first and last contraction were calculated, as were the force–time product of all contractions, measured as tension-time index (TTI), and ratings of perceived exertion (RPE).

**Results:**

Individuals with ME/CFS demonstrated smaller dO_2_Hb and dHHb than controls. However, after adjusting for TTI and change in total hemoglobin (delta (d)tHb), differences in dO_2_Hb and dHHb were reduced, with large overlapping variances. RPE was significantly higher for cases than controls, particularly at rest.

**Conclusions:**

Relative to controls, participants with ME/CFS demonstrated higher RPE, lower TTI, and reduced dO_2_Hb and dHHb during repetitive handgrip exercise, although considerable variance was observed. With further study, submaximal exercise testing may prove useful for stratifying patients with a lower propensity for inducing PEM, and have the ability to establish baseline intensities for exercise prescription.

## Background

Myalgic Encephalomyelitis/Chronic Fatigue Syndrome (ME/CFS) is a debilitating illness of unknown etiology characterized by profound fatigue that is markedly exacerbated by physical or mental activity. Over one million Americans experience symptoms compatible with ME/CFS and the Canadian Community Health Survey indicates that an estimated 350,000 Canadians report a diagnosis of the syndrome [1–3]. Furthermore, those with ME/CFS consistently rank as having more unmet medical and homecare needs, less food security, more marginalization, more need of help with tasks, greater difficulty in social situations, less ability to work, and lower personal income than those with most other chronic diseases [3–5].

Many studies have reported reduced exercise capacity in individuals with ME/CFS compared to healthy controls [6–10]. Indeed, a recent systematic review reported that more high quality studies encompassing a greater number of study subjects found decreased physiological exercise capacity in individuals with ME/CFS compared to the number and size of studies reporting no difference [9], although publication bias may explain these findings.

A hallmark of ME/CFS is a distinctive post-exertional malaise (PEM), also referred to as a symptom flare or “crash”, which occurs following physical or cognitive exertion and can last from days to weeks. The PEM response can occur following testing of ME/CFS patients’ exercise capacity, particularly after the maximal exercise testing protocols commonly used in ME/CFS, in which an individual performs exercise to the point of exhaustion [11–13].

Submaximal exercise testing, in which individuals perform at a workload below their maximum capacity, may be able to distinguish between cases with ME/CFS and healthy controls or to stratify individuals with ME/CFS, with the benefit of decreasing the risk of PEM following testing. Submaximal testing may also reach a larger group of patients, as some individuals may not be able to perform a maximal exercise test [14, 15] and the ethics of using maximal testing in this group of patients has been questioned [16]. Additionally, submaximal testing may be useful in screening for metabolic myopathies, such as mitochondrial disease, that may provide alternative explanations for ME/CFS patients’ symptoms.

Few studies have investigated ME/CFS using submaximal exercise testing and the conclusions they have drawn are inconsistent. Both Nijs et al. and Keller et al. investigated submaximal exercise testing’s ability to predict the results of maximal testing [17, 18]. Nijs et al. found that submaximal oxygen uptake, defined as 75% of the age-predicted heart rate, was correlated with peak oxygen uptake, although the correlation was not strong enough for prediction of peak oxygen uptake for clinical purposes. However, Keller et al. concluded that submaximal testing could not predict the results of a maximal test, particularly in patients experiencing PEM. Other experiments investigated submaximal testing using multiple types of exercise—exercise cycle, foot pedal, and elbow flexors. Using the pedal test, ME/CFS patients displayed significantly lower recovery rates for tissue oxygen saturation [19], whereas the cycle and handgrip tests suggested that only perceived exertion differed between ME/CFS cases and controls [20, 21]. To date, no studies have investigated the wrist flexors and extensor muscle oxygenation during repetitive handgrip exercise in ME/CFS patients.

Like pulse oximetry, near-infrared spectroscopy (NIRS) is a non-invasive technique for measuring oxygen saturation. Whereas pulse oximetry estimates arterial blood oxygenation, the more sophisticated NIRS technology enables tissue measurements of both oxygenated hemoglobin (O_2_Hb) and deoxygenated hemoglobin (HHb). Total hemoglobin (tHb) can be determined from the sum of O_2_Hb and HHb, providing a surrogate measure of local blood volume in muscle beneath the NIRS probes [22]. The procedure required to perform NIRS is simple with limited equipment required, making replication of our procedure by other laboratories straightforward.

It has been hypothesized that exercise testing might aid in the diagnosis of ME/CFS if suspected patients’ exercise outcomes are reliably distinct from those of healthy individuals [13]. Even if exercise testing proved not to have diagnostic utility, it may also be useful in characterizing a patient’s illness—for example, assessing the disability level of ME/CFS patients and providing a baseline against which a clinician can prescribe and evaluate an exercise and physical activity prescription. This study is the first of its kind to use NIRS to compare O_2_Hb and HHb in wrist flexors and extensor muscles during a submaximal, repetitive handgrip test in patients with ME/CFS and healthy controls. The objective was to identify different patterns of tissue oxygen utilization in individuals with ME/CFS compared to healthy controls, which would provide evidence that the poor exercise tolerance and rapid fatigue seen in ME/CFS is due to peripheral muscle dysfunction. We hypothesized that ME/CFS patients’ perceived exertion would be higher, and that their total workload, as quantified by the tension-time index (TTI), would be lower during the exercise protocol as compared to healthy controls. We further hypothesized that the decrease in O_2_Hb normally observed during submaximal repetitive workloads would be attenuated in patients with ME/CFS.

## Methods

### Recruitment

All participants were recruited as part of the Chronic Complex Diseases (CCD) study at the University of British Columbia, a case–control study designed to compare participants with chronic complex diseases using multiple platforms, from metagenomics and transcriptomics to exercise testing. Each participant with a physician-based diagnosis of ME/CFS fitting the Canadian case definition [23]—the most specific case definition identified at the time of study inception—was compared to one healthy person matched on gender and age within 5 years. To facilitate comparison with other studies, participants were also classified as to whether they met the Fukuda (CDC) case definition [24]. Participants were members of the general public who learned of the study through postings in newspapers and on websites or through physician referrals, and were recruited between 2012–05–23 and 2014–04–23. If participants had no exclusion criteria (age <19 years, unable to understand English, another diagnosed medical condition that fully explains symptoms, or antibiotic therapy in the last month), they were taken for pre-screening clinical assessment and documentation. All participants signed informed consent forms that were approved by the ethics board. The study aimed to recruit 25 cases with ME/CFS and 25 healthy controls, a number sufficient to identify a significant odds ratio of 20 between cases with ME/CFS and healthy controls, and chosen with the larger metagenomics arm of the study in mind, which is not described here.

Pre-screening included verification of the disease diagnosis and collection of age, height, weight, blood pressure, heart rate and ethnicity for each participant, as well as laboratory tests for hemoglobin, ferritin and acyl carnitine, all reported as part of this study. Additionally, pre-screening collected details of self-reported demographics and histories, including completion of the Fatigue Severity Scale (FSS) [25] and a physician review of systems, which involved counting the number of tender points to indicate level of body pain. Further laboratory blood screening tests were also performed. Orthostatic intolerance was determined by observing any of the following between sitting and standing: decrease in systolic blood pressure of 20 mmHg or more, decrease in diastolic blood pressure of 10 mmHg or more, or increase in heart rate of 30 BPM or more.

### Exercise testing protocol

#### Experimental design

A repetitive handgrip protocol was performed based on the protocol of Jeppesen et al. [26], and NIRS was used to monitor relative changes in O_2_Hb, HHb and tHb levels, as described previously [27, 28]. Before beginning the exercise test, all participants were screened using the AHA/ACSM Health/Fitness Facility Preparticipation Screening Questionnaire [29].While in the sitting position with the arms supported on a table, skinfold thickness was measured over the optode sites at the midpoint of the muscle belly, and NIRS optodes were applied to the wrist extensors and flexors groups, bilaterally. Resting blood pressure, heart rate and Borg’s rating of perceived exertion (RPE) [30] were assessed at baseline. Baseline monitoring of NIRS was performed for 5 min. The maximal voluntary contraction (MVC) for a handgrip was determined for the dominant arm (self-reported as the hand the participant wrote with) by performance of three MVCs. Forty percent of the MVC was calculated, which was the target force for the submaximal repetitive handgrip protocol. After a 5 min rest period, the participant performed repetitive handgrip contractions for 3 min; the participant was instructed to target 40% of the MVC at a contraction rate of 30 per minute and a duty cycle of 1:1. The last eight control participants performed a second repetitive handgrip protocol on the contralateral hand at 30% of the MVC in order to better match the absolute force produced by the cases with ME/CFS. This was preceded by measures of the MVC on the contralateral arm followed by a repetitive handgrip protocol at 30% of this MVC. At the end of the handgrip protocol, vital signs and RPE were repeated and the participants were instructed to sit at rest for an additional 5 min.

#### Repetitive handgrip protocol

A grip force transducer was connected to a PowerLab data acquisition unit that displayed output using LabChart software (ADInstruments, Colorado Springs, CO) on a computer monitor. Real-time visual feedback via the monitor display was provided to participants during the MVC and submaximal contractions. After three MVCs were performed within 5%, the participant rested for 5 min and then began the 3 min repetitive handgrip test [31]. RPE was quantified using a modified Borg scale, recorded before and immediately after completion of the test. The participants were required to choose a number between 0 and 10, anchored by “nothing at all” and “very, very severe”, respectively [30]. The target for the submaximal contractions was cued by observation of a computer monitor that displayed each contraction with a marker at 40% of the MVC for all subjects and at 30% of the MVC for the second test of the last eight control subjects. The repetition rate and duty cycle were cued by an audio recording that repetitively stated “contract-relax” for a 3 min period for a total of 90 contractions. One of the investigators provided verbal feedback to coach participants about the timing and force of the contractions in order to match the duty cycle and the 40 or 30% MVC target. The force–time product, termed the tension-time index (TTI), was calculated as the integral of all contractions by the LabChart software in Newton-seconds.

#### Near infrared spectroscopy (NIRS)

Muscle oxygenation and hemodynamics of the left and right flexor digitorum and extensor carpi radialis brevis (ECRB) were monitored continuously using a four-channel continuous-wave NIRS device (Oxymon Mk II, Artinis, The Netherlands). This device emits two wavelengths in the near infrared spectrum, 760 and 850 nm, that are preferentially absorbed by HHb and O_2_Hb, respectively. The principle of NIRS and calculation of NIRS-derived parameters have been described previously [32–34]. In brief, NIRS software contains algorithms based on the Beer-Lambert law [35] that calculate the absorbance of chromophores (O_2_Hb and HHb) based on the emitted reflected and received light and the absorbance is stated in arbitrary units (AU). Fluctuations in chromophore absorbance coincide with intermittent contractions, and hence, compression and release of the vasculature in the exercising muscle. Because of these fluctuations, the change in O_2_Hb [delta (d) O_2_Hb] and HHb (dHHb) chromophore concentration was calculated from the midpoint of the first contraction to the midpoint of the last contraction. The peak and nadir values of these contractions were determined in Oxysoft to four decimal places. The average of these two values was defined as the midpoint. The sum of O_2_Hb and HHb was also used to provide a measure of tHb, a surrogate for blood volume beneath the optode site. Skinfold thickness was measured prior to placement of NIRS optodes using a skinfold caliper in order to ensure that the adipose tissue covering the muscles was less than 10 mm. After the skin was cleaned with alcohol, four NIRS optodes were placed directly on the skin over the left and right flexor digitorum and ECRB. Position was standardized using anatomical landmarks and palpitation of the muscle belly. Optodes were fixed in place with double-sided sticky circular adhesives (3M™ Double-Stick Discs) and further secured with strips of low allergy adhesive tape (Hypafix^®^). Continuous recording of NIRS measures were performed throughout the rest periods, MVCs, and grip protocols.

### Statistical analysis

Statistical analyses comparing cases with ME/CFS to healthy controls were performed using STATA V12.1. Due to the non-normal distribution of the data, univariate analysis was performed using Fisher’s Exact Test to compare categorical variables and Mann–Whitney rank sum tests for continuous and ordinal variables. Subgroups were compared using a Kruskal–Wallis test and correlation between two continuous variables measured using Spearman’s rank correlation. Initial adjustment of the NIRS output for TTI and dtHb was performed by division of dO_2_Hb and dHHb by TTI or dtHb. Multivariate analyses were performed using linear regression with dummy variables for categorical variables, and residuals were confirmed to be normally distributed using the Shapiro–Wilk normality test. Curves resulting from exercise testing were depicted using a lowess smoother. Lowess carries out a locally weighted regression of the y variable (O_2_Hb or HHb) on the x variable (time) to display the graph as a smooth curve. This enabled visual comparisons among participants that would have been masked by the participant curves showing the usual fluctuations in O_2_Hb and HHb that occur during repetitive contraction and relaxation of the wrist flexors. Delta (d)O_2_Hb and dHHb were considered the primary outcome variables.

## Results

The CCD study pre-screened 102 people and excluded 52:24 with another diagnosis (e.g., sleep apnea, hypothyroidism, etc.), three who did not meet the case definitions, 11 that declined to participate, and 14 eligible patients that could not be matched to a control. The final overall study cohort comprised 25 cases with ME/CFS and 25 healthy controls. Of these, 22 ME/CFS cases and 21 healthy controls performed the repetitive handgrip protocol for the CCD exercise testing sub-study. Due to the underrepresentation of males amongst exercise testing participants, four male CFS cases and four male healthy controls were excluded from analysis to avoid confounding from pooling gender data. Additionally, one ME/CFS case that was subsequently diagnosed with sleep apnea and another that exhibited considerable muscle spasm and was unable to complete the protocol were excluded, as well as one healthy control with arthritis in their hand. The final cohort for analysis included 16 ME/CFS cases and 16 healthy controls, all female and largely Caucasian, and age-matched within a 5-year strata (Table 1). All cases and no healthy controls fit both the Canadian case definition and the CDC Fukuda definition of ME/CFS (Table 1).

**Table 1:** Characteristics of study participants

	ME/CFS cases (N = 16)	Healthy controls (N = 16)	*P* value* case vs. control*
Age (years)	55 (46.5;62.5)	54 (49.5;65)	0.76
BMI (kg/m^2^)	25.1 (21.7;31.5)	20.8 (19.2;23.9)	*0.03*
Skinfold thickness (mm)**	8 (7;10)	7 (5;10)	0.49
Ethnicity (Caucasian vs. other)	15 (94%)	13 (81%)	0.60
Fukuda definition	16 (100%)	0 (0%)	*<0.0005*
Fatigue Severity Scale	61 (56.5;63)	17 (13.5;21.5)	*<0.0005*
Orthostatic intolerance	1 (6.3%)	4 (25.0%)	0.3
Number of tender points	1 (0;6)	0 (0;0)	*0.009*
Hemoglobin (g/L)	134 (130;137)	131.5 (125.5;137.5)	0.47
Ferritin (μg/L)	43.5 (25;73.5)	43 (24;84)	1.00
Acyl carnitine (umol/L)	11.5 (8.3;15.0)	12.3 (8.9;14.0)	1.00

Values are N (%) or median (inter-quartile range (IQR). *BMI* Body Mass Index.

* P values are univariable exact test for discrete variables and rank sum for continuous variables. Significant *P* values are indicated in italics.

** Skinfold thickness measurement for 10 ME/CFS cases and 9 controls only.

Cases and controls did not differ significantly in ethnicity, hemoglobin, ferritin, or acyl carnitine levels; however, cases with ME/CFS had significantly higher scores on the FSS and a greater number of tender points, as well as greater body mass indexes (BMI) than controls, by a mean of 3.7 kg/m^2^. Despite the difference in BMI, skinfold differences over optode sites did not differ between groups in the 19 subjects for which it was recorded.

Fifteen of 16 cases with ME/CFS and all healthy controls completed the test with contractions for greater than 170 s (Table 2). The median TTI for ME/CFS cases was 26% lower than that of controls (*P* = 0.0008). Borg’s RPE was significantly higher both at rest and at the end of the repetitive handgrip protocol in the cases versus controls (*P* < 0.0001 and 0.001, respectively) (Table 2). Interestingly, the most significant difference in Borg’s RPE was at rest, when most cases with ME/CFS rated a level of exertion from simply attending the study, whereas most controls rated resting perceived exertion levels as “0—nothing at all”.

**Table 2:** Results of exercise test by participant

	CFS cases (N = 16)	Healthy controls (N = 16)	*P* value case vs. control
Test length (s)	177.1 (176.1;177.5)	176.3 (175.9;177.4)	0.60
TTI (Newton-seconds)	11,388 (8,463;13,934)	15,382 (12,921;16,974)	*0.008*
Borg at rest*	1.5 (1;2)	0 (0;0)	*<0.0005*
Borg at end*^,^**	3 (3;4)	2.5 (1.5;3)	*0.01*
dHHb (AU)	4.3 (3.4;10.4)	15.3 (9.1;24.2)	*0.008*
dHHb/dtHb (AU)	0.8 (0.6;1.5)	1.9 (1.2;3.3)	*0.04*
dHHb/TTI (AU/Newton-seconds)	0.0004 (0.002;0.001)	0.0009 (0.0006;0.002)	0.06
dO_2_Hb (AU)	0.2 (−4.2;1.4)	−6.7 (−12.9;−3.6)	*0.004*
dO_2_Hb/dtHb (AU)	0.2 (−0.5;0.4)	−0.9 (−2.3;−0.2)	*0.04*
dO_2_Hb/TTI (AU/Newton-seconds)	0.00002 (−0.0005;0.0001)	−0.0005 (−0.001;−0.003)	*0.02*
dtHb (AU)	5.2 (2.1;6.9)	9.8 (0.8;13.6)	0.31
dtHb/TTI (AU/Newton-seconds)	0.0005 (0.0002;0.0008)	0.0006 (0.00006;0.0009)	1.000

Results are N (%) for discrete variables or Median (IQR) for continuous and ordinal variables. *dO*_*2*_ *Hb* Delta-oxy-hemoglobin, *dHHb* delta-deoxy-hemoglobin, *dtHb* delta-total hemoglobin, *AU* arbitrary unit and *TTI* tension-time index.

*P* values are univariate exact test for discrete variables and rank sum for continuous and ordinal variables. Significant *P* values are indicated in italics. Confidence intervals not provided due to non-parametric analyses.

* Ordinal variable

** One CFS case did not report Borg at 3 min due to lightheadedness and the need to quickly lie supine.

**Figure 1:**
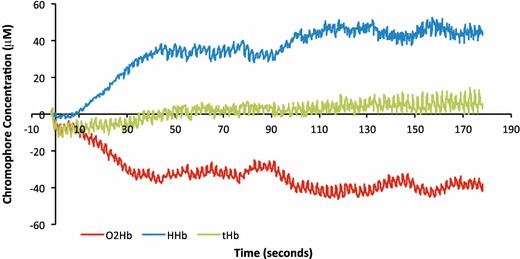
Changes in oxygenated hemoglobin [arbitrary units (AU)], deoxygenated hemoglobin (AU), and total hemoglobin (AU) of the right flexor digitorum during repetitive contractions of 3 min duration in one representative subject. Note the repetitive fluctuations that coincide with the intermittent contractions and hence compression and release of the vasculature in the exercising muscle.

**Figure 2:**
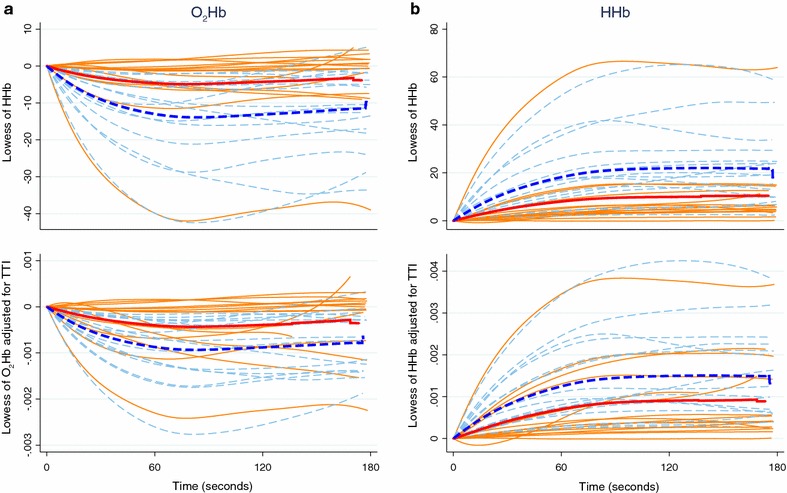
**a** Oxygenated hemoglobin levels throughout test before adjusting for tension-time index (TTI) (Newton-seconds) (*upper panel*) and after adjusting for TTI (*lower panel*). **b** Deoxygenated hemoglobin levels [arbitrary units (AU)] throughout test before adjusting for TTI (*upper panel*) and after adjusting for TTI (*lower panel*). *Orange* Myalgic Encephalomyelitis/Chronic Fatigue Syndrome (ME/CFS), *pale blue dash* healthy control, *red bold* mean ME/CFS and *royal blue bold* mean healthy control. Curves were smoothed using lowess-smoother.

**Figure 3:**
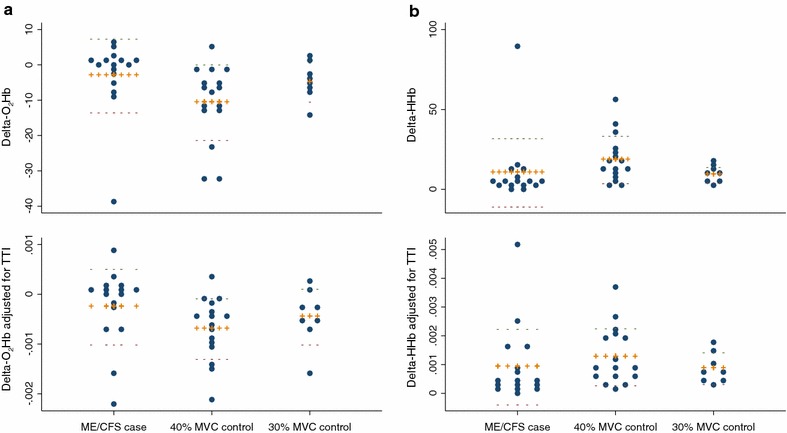
**a** Delta-oxygenated hemoglobin [arbitrary units (AU)] values for cases, controls, and 30% maximal voluntary contraction (MVC) controls, before (*upper panel*) and after (*lower panel*) adjusting for tension-time index (TTI) (Newton-seconds). **b** Delta-deoxygenated hemoglobin values before (*upper panel*) and after (*lower panel*) adjusting for TTI. *Orange crosses* mean, *green dash* mean plus standard deviation and *purple dash* mean minus standard deviation.

Figure 1 shows an example of the chromophore changes of a single participant during the handgrip protocol—a rapid decrease in oxygenated hemoglobin and corresponding increase in deoxygenated hemoglobin, and a gradual increase in total hemoglobin/local blood volume. Lowess-smoothed levels of O_2_Hb and HHb throughout the test for individual participants are shown in Figure 2, while Figure 3 summarizes the change, or delta, in each individual’s O_2_Hb and HHb between the test start and end.

Comparing the change in O_2_Hb and HHb between test start and end revealed that cases with ME/CFS displayed a smaller dO_2_Hb (*P* = 0.004) and dHHb (*P* = 0.008) compared to healthy controls (Table 2, Figure 3 upper panels). The significant differences remained after adjusting for dtHb, a surrogate for muscle blood volume (*P* = 0.04) and dtHb did not differ between groups (*P* = 0.31). After adjusting for TTI, the difference between cases and controls in dHHb (Figure 3b lower panel) was reduced (*P* = 0.06). Similarly after adjusting for TTI the difference in dO_2_Hb between cases and controls was reduced (Figure 3a lower panel), but remained significant at *P* = 0.02. However, considerable overlap between dO_2_Hb values among cases and controls was observed, particularly after TTI adjustment (Table 2; Figure 3a). Similar results were obtained when the area under the curve for O_2_Hb and HHb throughout the experiment was used as the outcome measure, with the difference between cases and controls reduced after adjusting for TTI (data not shown).

Since the range of symptoms within ME/CFS may vary, we also considered whether three measures that differ amongst ME/CFS sufferers were correlated with oxygen utilization: level of fatigue, body pain and orthostatic intolerance. We were unable to find a significant correlation between any of these measures and dO_2_Hb or dHHb within ME/CFS cases in our study (Table 3). We also considered whether RPE may be correlated with level of body pain, however, no significant correlations were found between level of body pain and RPE at rest (*P* = 0.6) or RPE at 3 minutes (*P* = 1.0). Finally we hypothesized that lower TTI may be related to dysautonomia in ME/CFS patients, which we investigated through orthostatic intolerance. Since only four ME/CFS patients had orthostatic intolerance, statistical significance was not achieved (*P* = 0.1), but it was notable that ME/CFS cases with orthostatic intolerance had lower median values for TTI (8,778 vs. 11,436 Newton-seconds) and two of the four ME/CFS individuals with orthostatic intolerance had the two lowest TTI values seen in the study.

**Table 3:** Relation of Fatigue Severity Scale, number of tender points and orthostatic intolerance to dO_2_Hb and dHHb

	FSS		Number of tender points		Orthostatic intolerance		
	Spearman’s rho	*P*	Spearman’s rho	*P*	Yes*	No*	*P*
dO_2_Hb (AU)	0.198	0.5	−0.2293	0.4	−0.4 (−5.0;0.52)	0.6 (−4.2;2.3)	0.9
dHHb (AU)	−0.2385	0.4	0.0785	0.8	5.3 (4.3;10.0)	4.2 (2.7;12.4)	0.9

N = 16

*P* value are Spearman’s rank correlation coefficient for FSS and number of tender points and rank sum for orthostatic intolerance.

*FSS* Fatigue Severity Scale, *dO*_*2*_ *Hb* delta-oxy-hemoglobin, *dHHb* delta-deoxy-hemoglobin and *AU* arbitrary unit.

* Median (IQR).

To account for the effect of other variables on dO_2_Hb, we entered ME/CFS case versus healthy control, TTI, dtHb, Borg at rest, age, BMI, ethnicity, FSS, orthostatic intolerance, number of tender points, ferritin, and hemoglobin, into a regression model with dO_2_Hb as the dependent variable. The only factors that were significant predictors of dO_2_Hb were dtHb (*P* = 0.0125), BMI (*P* = 0.025) and age, which was only just a significant predictor at the 0.05 level (*P* = 0.049) (Table 4). Similarly, substituting dHHb as the dependent variable also found dtHb and BMI to be significant predictors of dHHb after adjusting for the above variables (*P* = <0.0005 and 0.025 respectively) (data not shown).

**Table 4:** Multivariable regression of dO_2_Hb

Factor	*P* value	95% Confidence interval
ME/CFS case vs. healthy control	0.27	−5.22:17.88
Tension-time index (Newton-seconds)	0.20	−0.0017:0.00038
dtHb (AU)	*0.025*	−0.78:−0.59
Borg RPE at rest	0.76	−3.43:2.52
Age (years)	*0.049*	−0.82:−0.0025
BMI (kg/m^2^)	*0.025*	−0.12:1.57
Ethnicity (caucasian vs other)	0.71	−13.62:9.40
Fatigue Severity Scale	0.36	−0.86:0.33
Orthostatic intolerance	0.17	−19.10:3.59
Number of tender points	0.38	−0.72:1.82
Hemoglobin (g/L)	0.87	−0.41:0.48
Ferritin (μg/L)	0.52	−0.028:0.054

*BMI* Body mass index, *RPE* rating of perceived exertion and *AU* arbitrary unit.

Significant *P* values are indicated in italics.

The range of dO_2_Hb values was similar for cases with ME/CFS and controls, at −38.9–6.4 and −32.4–5.4 respectively (Figure 3), with respective standard deviations of 10.7 and 10.5. However, one ME/CFS case significantly extended the range with an uncharacteristically large delta. This participant was in the 50–55 year age stratum, of Caucasian ethnicity, and their only distinctive characteristic was a low BMI (15.6 kg/m^2^). We divided ME/CFS cases into three groups according to their dO_2_Hb: (1) “Low” dO_2_Hb <−5 (N = 4); (2) “Medium” −5 ≤ dO_2_Hb < 5 (N = 10); and (3) “High” dO_2_Hb ≥5 (N = 2). There were no significant differences in age, BMI, ethnicity, fatigue severity, number of tender points, orthostatic intolerance, hemoglobin, ferritin, or acyl carnitine between the subgroups.

In order to better match the absolute force produced by the cases with ME/CFS, a subset of eight controls completed a duplicate test at 30% MVC. Controls who completed the test at 30% MVC did not significantly differ from cases in any of the variables investigated in Table 1 (data not shown); however, it must be noted that power to detect differences between 30% cases and controls was low, due to the small number of controls who repeated the test. Median test length for ME/CFS cases and 30% MVC controls did not differ (*P* = 0.81), nor did TTI (*P* = 1.00), unlike the comparison between cases with ME/CFS and 40% MVC healthy controls. Comparison of cases with ME/CFS to the eight controls who performed the test at 30% MVC removed significant differences in dO_2_Hb (*P* = 0.20) and dHHb (*P* = 0.11), before and after adjusting for TTI (dO_2_Hb *P* = 0.33, dHHb *P* = 0.20) and dtHb (dO_2_Hb, *P* = 0.076, dHHb *P* = 0.076).

Two participants with ME/CFS suffered adverse reactions to the study, presenting as muscle spasm and hypotension. Additionally, three ME/CFS participants with particularly low levels of HHb throughout the study were referred to a metabolic clinic. All three have tested negative for mitochondrial disease.

## Discussion

We identified a significant difference in oxygenated and deoxygenated hemoglobin levels between cases with ME/CFS and healthy controls during a three-minute submaximal handgrip exercise testing procedure. However, after adjusting for total hemoglobin or the force at which the procedure was performed, these differences were reduced. Furthermore, particularly after adjusting for TTI, we observed significant overlap in dO_2_Hb and dHHb between cases with ME/CFS and controls, and after adjusting for multiple factors in a multivariable model, case versus control did not have a significant effect on dO_2_Hb or dHHb, indicating that neither measure is appropriate for diagnostic testing.

Although our observations indicate that tissue oxygenation levels during submaximal exercise testing may not be useful in differentiating cases with ME/CFS from healthy controls, the large range in deoxyhemoglobin levels seen in participants with ME/CFS suggested that submaximal exercise testing could conceivably be used to subtype individuals with ME/CFS, perhaps into low, medium and high categories. Such deoxyhemoglobin-based subtyping could be used in determining baselines for exercise prescription and screening for adverse responses. Additionally, submaximal exercise testing may flag patients with metabolic myopathies [36, 37] whose symptoms resemble those of ME/CFS. We observed extremely low HHb values during exercise testing in three study participants; they were referred for further testing, however, genetic testing for inherited mitochondrial disease revealed no abnormalities.

Although we did not observe substantial differences in oxygen saturation between cases and controls, other differences were apparent. ME/CFS cases performed the procedure at significantly lower TTI than healthy controls, suggesting that the force at which the handgrip was squeezed is one possible explanation for the difference in unadjusted blood oxygenation levels. However, after adjusting for TTI alone, differences in dO_2_Hb and dHHb remained, albeit reduced. Reduced TTI may also be related to patients with dysautonomia, as two ME/CFS patients in this study with orthostatic intolerance had particularly low TTI however, further studies would be required to confirm this. An alternative explanation for the lower TTI is reduced redistribution of cardiovascular output to exercising muscles resultant from impairment or lack of fitness in ME/CFS patients. Reduced fitness may result from patients with ME/CFS’s aversion to physical activity due to fear of fatigue. This aversion increasingly perpetuates deconditioning, similar to the pain catastrophizing theory [38]. We chose a procedure that used the flexor digitorum, since this muscle is regularly activated while using computers and other devices, even in a sedentary state, so is less likely to be affected by deconditioning. Indeed, preservation of upper versus lower extremity musculature has been shown in hospitalized patients [39] and other chronic diseases such as COPD [40]. Furthermore, handgrip grip procedures have been shown to correlate with cardiopulmonary exercise testing using a cycle [41].

Our choice of protocol also meant that our test was less exhausting than other submaximal tests using methods such as an exercise cycle [20]. Even so, cases with ME/CFS perceived a greater level of exertion during the test, as measured by the Borg RPE scale. In fact, the median modified Borg rating for ME/CFS cases after the procedure was three and the highest seven, which corresponds to “very severe”. This difference in perceived exertion has previously been observed in ME/CFS patients and may be explained by central perception. Wallman et al. 2007 suggested that ME/CFS patients may have augmented afferent feedback, making them feel a gain in perceived effort. Furthermore, the procedure was tolerated very poorly for two individuals with ME/CFS, suggesting that even submaximal testing may carry a risk of PEM.

Because this was a sub-study within a larger investigation of complex chronic disease, the case–control sample sizes that were appropriate for the other study aims resulted in some limitations with respect to the exercise testing work. The small sample size meant that there was only ~50% power to detect a difference in dO_2_Hb between individuals with ME/CFS and healthy controls given the mean and standard deviations seen in this study. Therefore it is possible that we may have missed differences between groups, even more so in the subset of eight controls who repeated the procedure at 30% MVC. Despite these limitations, significant differences in dO_2_Hb were observed between groups, nonetheless, experiments using larger sample size would be beneficial to confirm our findings.

To our knowledge no studies have investigated gender differences in submaximal exercise testing in ME/CFS. However, maximal exercise testing has shown that women have lower exercise capacity, and therefore pooling gender data in studies with an unequal gender may confound results. [42] Due to the low numbers of male participants in our study, we therefore chose to remove their results from analysis, thus it is uncertain whether our findings are generalizable to male ME/CFS sufferers. Cases with ME/CFS and healthy controls were matched within a 5-year age stratum however, due to difficulties in recruiting, no further matching was made. ME/CFS cases had also significantly higher BMI than controls thus it would have been beneficial to match for BMI. However, skinfold thickness, which may have an impact on measurement of blood oxygenation, did not differ between cases and controls in the 19 individuals for which it was recorded. It may also have been beneficial to match participants on fitness levels, as seen in other studies [19–21], and additional measures that may affect results, such as blood flow, could also have been collected during the study and considered in multivariable analysis. However, the effect of blood flow was likely captured in dtHb, which proved to be a significant predictor of blood oxygenation in our study. Finally, in future studies, it may be informative to repeat the test after 24 h. Preliminary findings suggest that some patients with ME/CFS experience a drop in anaerobic threshold that can be measured on the second of two daily maximal cardiopulmonary exercise tests, a finding not seen in healthy, deconditioned controls [11–13]; however, no studies to date investigating ME/CFS using submaximal testing have compared results on subsequent days.

## Conclusions

This submaximal exercise testing protocol revealed attenuated changes in oxygenated and deoxygenated hemoglobin, which may be attributable to poor exercise tolerance and rapid fatigue seen in ME/CFS compounded by decreased fitness. Because of the variable responses among participants, it does not provide a clear distinction between cases with ME/CFS and healthy controls and is therefore not a useful diagnostic marker. However, testing did reveal a disproportionate level of perceived exertion and lower force production in cases with ME/CFS, and in two cases, adverse responses to low levels of exercise. The repetitive handgrip protocol may be useful to screen individuals for adverse responses and other conditions, including mitochondrial disease. It may also be useful for stratifying ME/CFS sufferers to determine appropriate levels of exercise prescription.

## Abbreviations

ME/CFS: Myalgic Encephalomyelitis/Chronic Fatigue Syndrome
PEM: post-exertional malaise
NIRS: near-infrared spectroscopy
O_2_Hb: oxygenated hemoglobin
HHb: deoxygenated hemoglobin
dO_2_Hb: delta oxygenated hemoglobin
dHHb: delta deoxygenated hemoglobin
TTI: tension-time index
RPE: rating of perceived exertion
dtHb: delta total hemoglobin
tHb: total hemoglobin
CCD: Chronic Complex Diseases
CDC: Center for Disease Control
FSS: Fatigue Severity Scale
MVC: maximal voluntary contraction
ECRB: extensor carpi radialis brevis
AU: arbitrary units
BMI: body mass index
IQR: inter-quartile range
mmHg: millimeter of mercury
BPM: beats per minute

## References

[1] Afari N, Buchwald D (2003). Chronic fatigue syndrome: a review. Am J Psychiatry.

[2] Reid S, Chalder T, Cleare A, Hotopf M, Wessely S (2000). Chronic fatigue syndrome. BMJ.

[3] Parlor M (2009) Profile and impact of 23 chronic conditions in the 2005 canadian community health survey. Quest—National ME/FM action network. Spring Summer 2009(80): 1–16

[4] Reynolds KJ, Vernon SD, Bouchery E, Reeves WC (2004). The economic impact of chronic fatigue syndrome. Cost Eff Resour Alloc C/E.

[5] Solomon L, Nisenbaum R, Reyes M, Papanicolaou DA, Reeves WC (2003). Functional status of persons with chronic fatigue syndrome in the Wichita, Kansas, population. Health Qual Life Outcome.

[6] De Becker P, Roeykens J, Reynders M, McGregor N, De Meirleir K (2000). Exercise capacity in chronic fatigue syndrome. Arch Intern Med.

[7] Cook DB, Stegner AJ, Nagelkirk PR, Meyer JD, Togo F, Natelson BH (2012). Responses to exercise differ for chronic fatigue syndrome patients with fibromyalgia. Med Sci Sports Exerc.

[8] Fulcher KY, White PD (2000). Strength and physiological response to exercise in patients with chronic fatigue syndrome. J Neurol Neurosurg Psychiatry.

[9] Nijs J, Aelbrecht S, Meeus M, Van Oosterwijck J, Zinzen E, Clarys P (2011). Tired of being inactive: a systematic literature review of physical activity, physiological exercise capacity and muscle strength in patients with chronic fatigue syndrome. Disabil Rehabil.

[10] Inbar O, Dlin R, Rotstein A, Whipp BJ (2001). Physiological responses to incremental exercise in patients with chronic fatigue syndrome. Med Sci Sports Exerc.

[11] Vanness JM, Snell CR, Strayer DR (2003). Dempsey Lt, Stevens SR. Subclassifying chronic fatigue syndrome through exercise testing. Med Sci Sports Exerc.

[12] Snell CR, Vanness JM, Strayer DR, Stevens SR (2005). Exercise capacity and immune function in male and female patients with chronic fatigue syndrome (CFS). In Vivo.

[13] Snell CR, Stevens SR, Davenport TE, Van Ness JM (2013). Discriminative validity of metabolic and workload measurements for identifying people with chronic fatigue syndrome. Phys Ther.

[14] Noonan V, Dean E (2000). Submaximal exercise testing: clinical application and interpretation. Phys Ther.

[15] Nijs J, Zwinnen K, Meeusen R, de Geus B, De Meirleir K (2007). Comparison of two exercise testing protocols in patients with chronic fatigue syndrome. J Rehabil Res Dev.

[16] Van Oosterwijck J, Nijs J, Meeus M, Lefever I, Huybrechts L, Lambrecht L (2010). Pain inhibition and postexertional malaise in myalgic encephalomyelitis/chronic fatigue syndrome: an experimental study. J Intern Med.

[17] Keller B (2014). Superior ability ot a two-day CPET protocol to detect functional impairment in ME/CFS compared to either a single CPET, a submaximal exercise test, or a VO_2_ prediction equation.

[18] Nijs J, Demol S, Wallman K (2007). Can submaximal exercise variables predict peak exercise performance in women with chronic fatigue syndrome?. Arch Med Res.

[19] McCully KK, Natelson BH (1999). Impaired oxygen delivery to muscle in chronic fatigue syndrome. Clin Sci.

[20] Wallman KE, Morton AR, Goodman C, Grove R (2004). Physiological responses during a submaximal cycle test in chronic fatigue syndrome. Med Sci Sports Exerc.

[21] Wallman KE, Sacco P (2007). Sense of effort during a fatiguing exercise protocol in chronic fatigue syndrome. Res Sports Med.

[22] Okamoto T, Kanazawa H, Hirata K, Yoshikawa J (2003). Evaluation of oxygen uptake kinetics and oxygen kinetics of peripheral skeletal muscle during recovery from exercise in patients with chronic obstructive pulmonary disease. Clin Physiol Funct Imaging.

[23] Carruthers BM, Jain AK, De Meirleir K, Peterson D, Klimas N, Lerner AM (2003). Myalgic encephalomyelitis/chronic fatigue syndrome: clinical working case definition, diagnostic and treatment protocols. J Chron Fatigue Syndr.

[24] Fukuda K, Straus SE, Hickie I, Sharpe MC, Dobbins JG, Komaroff A (1994). The chronic fatigue syndrome: a comprehensive approach to its definition and study. International Chronic Fatigue Syndrome Study Group. Ann Intern Med.

[25] Krupp LB, LaRocca NG, Muir-Nash J, Steinberg AD (1989). The fatigue severity scale. Application to patients with multiple sclerosis and systemic lupus erythematosus. Arch Neurol.

[26] Jeppesen TD, Quistorff B, Wibrand F, Vissing J (2007). 31P-MRS of skeletal muscle is not a sensitive diagnostic test for mitochondrial myopathy. J Neurol.

[27] Shadgan B, Guenette JA, Sheel AW, Reid WD (2011). Sternocleidomastoid muscle deoxygenation in response to incremental inspiratory threshold loading measured by near infrared spectroscopy. Respir Physiol Neurobiol.

[28] Abe K, Matsuo Y, Kadekawa J, Inoue S, Yanagihara T (1997). Measurement of tissue oxygen consumption in patients with mitochondrial myopathy by noninvasive tissue oximetry. Neurology.

[29] Thompson W, Gordon N, Pescatello L (2010) ACSM’s guidelines for exercise testing and prescription, 8th edn. Lippincott Williams & Wilkin, China

[30] Borg GA (1982). Psychophysical bases of perceived exertion. Med Sci Sports Exerc.

[31] Jensen TD, Kazemi-Esfarjani P, Skomorowska E, Vissing J (2002). A forearm exercise screening test for mitochondrial myopathy. Neurology.

[32] van der Sluijs M, Colier W, Houston R, Oeseburg B (1997). New and highly sensitive continuous-wave near-infrared spectrophotometer with multiple detectors. Proc SPIE.

[33] Grassi B, Quaresima V, Marconi C, Ferrari M, Cerretelli P (1999). Blood lactate accumulation and muscle deoxygenation during incremental exercise. J Appl Physiol.

[34] Tachtsidis I, Tisdall M, Leung TS, Cooper CE, Delpy DT, Smith M (2007). Investigation of in vivo measurement of cerebral cytochrome-*c*-oxidase redox changes using near-infrared spectroscopy in patients with orthostatic hypotension. Physiol Meas.

[35] Boushel R, Langberg H, Olesen J, Gonzales-Alonzo J, Bulow J, Kjaer M (2001). Monitoring tissue oxygen availability with near infrared spectroscopy (NIRS) in health and disease. Scand J Med Sci Sports.

[36] Meulemans A, Gerlo E, Seneca S, Lissens W, Smet J, Van Coster R (2007). The aerobic forearm exercise test, a non-invasive tool to screen for mitochondrial disorders. Acta Neurol Belg.

[37] Grassi B, Marzorati M, Lanfranconi F, Ferri A, Longaretti M, Stucchi A (2007). Impaired oxygen extraction in metabolic myopathies: detection and quantification by near-infrared spectroscopy. Muscle Nerve.

[38] Quartana PJ, Campbell CM, Edwards RR (2009). Pain catastrophizing: a critical review. Expert Rev Neurother.

[39] Hoenig HM, Rubenstein LZ (1991). Hospital-associated deconditioning and dysfunction. J Am Geriatr Soc.

[40] Castagna O, Boussuges A, Vallier JM, Prefaut C, Brisswalter J (2007). Is impairment similar between arm and leg cranking exercise in COPD patients?. Respir Med.

[41] Chen CH, Chen YJ, Tu HP, Huang MH, Jhong JH, Lin KL (2014). Benefits of exercise training and the correlation between aerobic capacity and functional outcomes and quality of life in elderly patients with coronary artery disease. Kaohsiung J Med Sci.

[42] Sargent C, Scroop GC, Nemeth PM, Burnet RB, Buckley JD (2002). Maximal oxygen uptake and lactate metabolism are normal in chronic fatigue syndrome. Med Sci Sports Exerc.

